# A Quantitative Approach for the Bone-implant Osseointegration Assessment Based on Ultrasonic Elastic Guided Waves

**DOI:** 10.3390/s19030454

**Published:** 2019-01-22

**Authors:** Benjamin Steven Vien, Wing Kong Chiu, Matthias Russ, Mark Fitzgerald

**Affiliations:** 1 Department of Mechanical & Aerospace Engineering, Monash University, Wellington Rd, Clayton 3800, Australia; wing.kong.chiu@monash.edu; 2 The Alfred Hospital, 55 Commercial Road, Melbourne 3004, Australia; M.Russ@alfred.org.au (M.R.); M.Fitzgerald@alfred.org.au(M.F.); 3 National Trauma Research Institute, 89 Commercial Road, Melbourne 3004, Australia

**Keywords:** acousto-ultrasonic, guided wave, structural health monitoring, osseointegration, implant, prosthesis

## Abstract

Quantitative and reliable monitoring of osseointegration will help further evaluate the integrity of the orthopaedic construct to promote novel prosthesis design and allow early mobilisation. Quantitative assessment of the degree or the lack of osseointegration is important for the clinical management with the introduction of prosthetic implants to amputees. Acousto-ultrasonic wave propagation has been used in structural health monitoring as well as human health monitoring but so far has not extended to osseointegrated implants or prostheses. This paper presents an ultrasonic guided wave approach to assess the osseointegration of a novel implant. This study explores the potential of integrating structural health monitoring concepts into a new osseointegrated implant. The aim is to demonstrate the extension of acousto-ultrasonic techniques, which have been widely reported for the structural health monitoring of engineering structures, to assess the state of osseointegration of a bone and implant. To illustrate this potential, this paper will report on the experimental findings which investigated the unification of an aluminium implant and bone-like geometry surrogate. The core of the test specimen is filled with silicone and wrapped with plasticine to simulate the highly damped cancellous bone and soft tissue, respectively. To simulate the osseointegration process, a 2-h adhesive epoxy is used to bond the surrogate implant and a bone-like structure. A series of piezoelectric elements are bonded onto the surrogate implant to serve as actuators and sensors. The actuating piezoelectric element on an extramedullary strut is excited with a 1 MHz pulse signal. The reception of the ultrasonic wave by the sensing elements located on the adjacent and furthest struts is used to assess the integration of this implant to the parent bone structure. The study shows an Osseointegration Index can be formulated by using engineering and acousto-ultrasonic methods to measure the unification of a bone and implant. This also highlights a potential quantitative evaluation technique regardless of bone-implant geometry and soft tissue damping.

## 1. Introduction

Since 1965, the osseointegration technique has been used in the area of dentistry and extended to orthopaedics and limb amputees in pioneering work by Bårenmark. Titanium and its alloys are commonly used as implants due to their excellent biocompatibility and superior mechanical properties. Nevertheless, the osseointegration process is complex, and many factors influence the formation of the bone at the implant surface [1,2]. Recent studies have shown that patients with osseointegrated prosthesis have improved mobility and better quality of life than patients with socket prosthesis [3,4,5,6]. However, previous studies reported that the currently available implants result in bone stress-shielding; loads are taken by the implant and shielded from distributing to the bone, and hence, causing bone loss which limits the implant longevity and degree of osseointegration [7,8,9].

The standard osseointegration for trans-femoral implants has two surgical procedure stages: (1) An implant is inserted into the femur; and later (2) an abutment is fitted to the implant [10] through the soft tissue cover to attach a prosthesis. The healing phase of osseointegration normally takes 6 to 12 months, and most recently the standard duration was shortened to 3 months. However, the time in rehabilitation can be further shortened. It is also possible to start immediately loading the femur in patients with healthier bone quality [11]. These patients would benefit greatly, physically and mentally, from an optimised and personalised rehabilitation time [10,11].

The osseointegration can be affected by a local infection usually occurring on the skin-implant interface. Any micromotion during the initial stage of osseointegration can cause a lack of unification. It is essential that the implant/prosthesis is gradually loaded until the implant can accept the full body weight as most of the current implants do not provide the initial stability for such loading during the first 12 months. Furthermore, patients are regularly evaluated on the prosthesis, mobility and quality of life in a monthly or yearly basis [2,10,12]; thus it is difficult to adequately predict the type of failures and severe trauma associated to the implant. Therefore, an evaluation technique of osseointegration is essential to understand the implant design and failures comprehensively.

More complex and advanced fixations and implants are studied and designed to promote shorter osseointegration and rehabilitation time by preventing bone stress-shielding and bone loss, and thus improving the quality of life [7,8,9,12,13,14,15,16,17,18,19,20,21,22]. Current bone and osseointegration assessments include radiographic study, clinical presentation, and diagnostic and nuclear imaging, which are known to be subjective and open to interpreter variability where its accuracy depends on the surgeon’s experience [16,23,24,25]. Previous studies have examined the level of osseointegration by using various proposed methods, such as X-ray examination, removal torque and push-out test [26,27,28]. However, due to their invasive nature and inaccuracy, these techniques could not be used to accurately assess the level of osseointegration. Recently, mechanical vibration analysis has been considered in the medical field and studies have used vibration analysis to assess the healing of a fractured femur and pelvis, and implant systems [29,30,31,32,33,34]. Ong et al.’s studies [31,33] extended engineering concepts to quantify the healing of fractured femur by using changes in the amplitude and frequency of vibrational modes. The ability to quantitatively evaluate the degree of osseointegration is important in clinical management as a timely surgical intervention can be initiated to ensure minimal disruption to the patient’s well-being. However, currently there is no applicable method for continuous quantification and monitoring.

Acousto-ultrasonic stress wave propagation is commonly used and actively researched as a non-destructive technique to characterise and assess metallic and composite structures for damage [35,36,37,38,39,40,41,42,43,44,45,46,47,48,49,50,51,52,53,54,55,56]. Furthermore, an array of sensors can be embedded in the structure to provide continuous structural health monitoring [38,41]. Guided wave propagations on cylindrical and thin-walled beam structures for structural health monitoring have also been investigated [48,50,51,57,58]. Studies have employed stress wave methods to assess fracture and bone quality by measuring guided/bulk wave velocity [23,48,59,60,61,62,63,64,65]. Previous studies have used acousto-ultrasonic approaches to potentially assess osseointegrated dental implants [35,66,67,68,69]. More recently, guided-wave techniques have been introduced to assess in the osseointegration of femur and implant design by Wang and Lynch [70,71]. Their work reported a decrease in energy of the longitudinal wave mode in a solid titanium rod implanted in a composite femoral bone.

The objective of this study is to first introduce the novel customisable implant design, which is ideally suited for sensor integration for structural integrity assessment. The study serves as an extension of our previous FE investigation [72] and will also describe a series of investigation into establishing a fundamental assessment model to monitor osseointegration of an implant design in the femur by using ultrasonic guided waves. This ultrasonic elastic guided wave method is based on measuring frequency response to determine the state of unification of the specimen. Furthermore, an index associated to the level of osseointegration for integrity assessment is formulated. This study intends to instigate and promote further development of smart human health monitoring design by incorporating engineering principles and methods for more informative and robust health care.

## 2. Background

### 2.1. Implant Design

There is currently no information about the long-term outcomes with the current developed implant systems and the early evaluation of the implants fundamentally provides important understandings to further optimise future designs for the later stage of osseointegration. The clinically approved osseointegrated leg prostheses (OILP) are: the Osseointegrated Prostheses for the Rehabilitation of Amputees (OPRA) system [11], the Integrated Leg Prosthesis (ILP) system [22] and the Compress^®^ device [19,20,21]. The OPRA system comprises of an intramedullary titanium screw while the ILP system involves a press-fit intramedullary implant. These large endoprosthesis implants are commonly used in arthroplasty as it provides good primary stability [16,73]. However, despite the advantages of OILP, their process requires a large amount of cancellous bone to be removed by intramedullary reaming. This indirect heating from reaming and bone removal process will negatively impact the osteogenic factor and, thus, resulting in loss of the essential bone substances. Clinical and numerical assessments have reported on bone resorption and stress-shielding in the near-prosthetic location, which potentially causes aseptic loosening and construct failure [7,8,9,17]. The Compress^®^ device, a complaint pre-stress fixation system, has been designed to tackle the issue of stress-shielding, which is accomplished using an implant system that applies constant compressive force across the bone-implant interface and with a smaller intramedullary component [19,20,21]. This small implant design greatly prompts osteogenic factor which is desirable for secondary stability in later stages of osseointegration. However, in some cases, it does not provide adequate stability to support physiologic loading in the early stages of osseointegration due to torsional overload [16].

**Figure 1:**
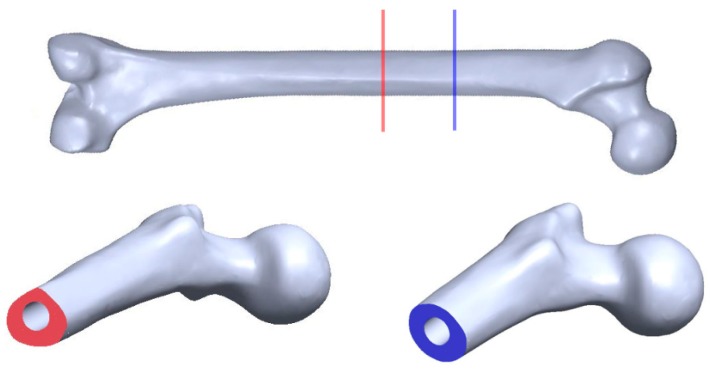
3D scanned bone showing triangular and circular cross-sectional areas at different anatomic locations.

**Figure 2:**
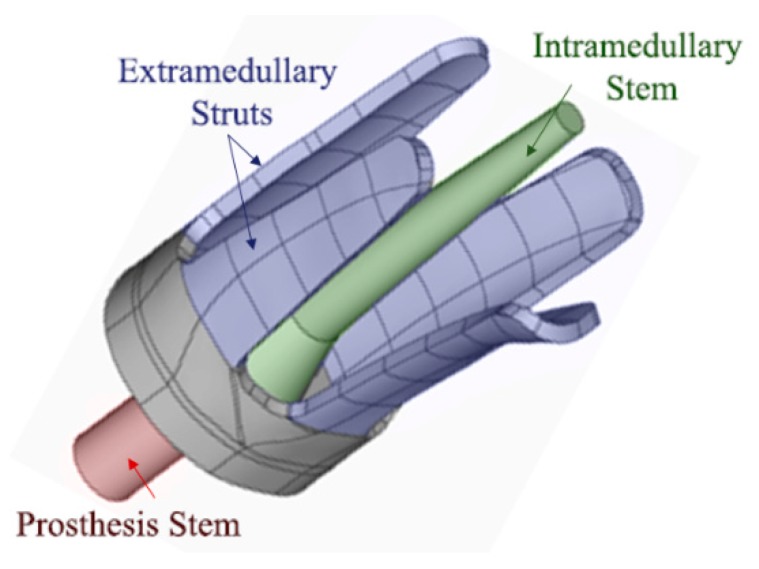
The novel customisable osseointegrated implant design introduced by Russ et. al. [2].

Amputations generally occur in different anatomic locations for each patient and no bone geometry is exactly same (refer to Figure 1). The current osseointegrated implant devices mainly rely on further bone alternation or removal of amputated bone for a good fit with the device. Russ, Fitzgerald and Chiu recently developed a new customisable osseointegration implant for long bones (Australian Patent No. 2017902308) [74]. This customised implant design will be installed without the need for reaming or bone removal. Russ et al. [16] reported on a novel osseointegration implant design which combines the extramedullary struts and intramedullary stem to ensure both initial and long-term stability, refer to Figure 2.

This customisable implant involves using CT imaging for a perfect fit, which bone substance is preserved since reaming is not required in the procedure, and Finite Element Analysis to optimise shape and stress distribution for each individual patient to minimise stress-shielding and bone loss. Lastly, this complex implant is then 3D-printed using biocompatible materials as one unitised structure for fast and accurate production without the need and complication of multicomponent assembly. In addition, this implant design can incorporate potential sensing technique by embedding sensors on the extramedullary struts and/or intramedullary stem. This paper is the first study to investigate the incorporation of acousto-ultrasonic methods on this particular novel implant design for continuous osseointegration monitoring.

### 2.2. Acousto-ultrasonic Concept for Osseointegration Index

In order to evaluate the degree of osseointegration, the acousto-ultrasonic methods and frequency analysis techniques, which have been used for SHM to describe the material mechanical properties [35,37,38,39,40,41,42,43], are considered. The acousto-ultrasonic evaluation has been studied in bone healing assessments, such as fractured bone healing, structural form, and osteoporosis, by utilising the wave propagation velocity and time-of-flight method [23,61,62,63]. Over the past decade, there is particular interest in stress wave propagation for quantitative analysis, and furthermore, guided waves propagation in bone has also been considered both computationally and in vivo experiments [59,64,65,70,71,75,76].

In structural health monitoring, a damage index in time or frequency domain is considered to quantify changes in some characteristics of the measured wave signal and some known parameters. Although various damage indices are used in the literature, the basic principle is to define a metric as the ratio between the scattered energy and the baseline energy of the time window [41,44,45,46,47]. This study incorporates the fundamental concept introduced by Lichtenwalner et al. [41], where the difference signals were used to determine a single metric for damage. This statistic was adopted by Wong et al. [34] for monitoring the fracture healing of an internally fixated pelvis by using vibration analysis. In this study, the proposed scheme uses the difference signal in the frequency domain and the concept of transfer energy to formulate an Osseointegration Index (O-Index), extending the previous findings by Wong et al. [34] and Ong et al. [31,33]. The purpose of using the difference time signal and normalising relative to the baseline signal serves to compare the O-Index between different sensors and specimens. The normalised O-Index (Equation (1)) is defined as the power of the difference signal between a frequency bandwidth relative to the baseline power, refer below:

(1) $$Osseointegration\;Index\;\left(T=T_{i}\right)\propto \int_{f_{1}}^{f_{2}}\frac{{\left|G\left(T,f\right)\right|}^{2}}{{\left|G_{0}\left(f\right)\right|}^{2}}\;df$$

$$G\left(T,f\right)=\int_{-\infty }^{\infty }g\left(T,t\right)e^{-i2\pi ft}dt\;\mathrm{and}\;G_{0}\left(f\right)=\int_{-\infty }^{\infty }g\left(T_{0},t\right)e^{-i2\pi ft}dt\;g\left(T,t\right)=g\left(T_{i},t\right)-g\left(T_{0},t\right)$$

where $g\left(T_{i},t\right)$ is the time signal at any cure time, $g\left(T_{0},t\right)$ is the time signal (baseline signal) at initial cure time $T_{0}=0$ min, g(T,t) is the difference in time signal relative to initial cure time, $G_{0}\left(f\right)$ and G(T,f) are the Fourier Transform of the baseline signal and difference in cure time signal, respectively, and $T_{i}\neq T_{0}$.

**Figure 3:**
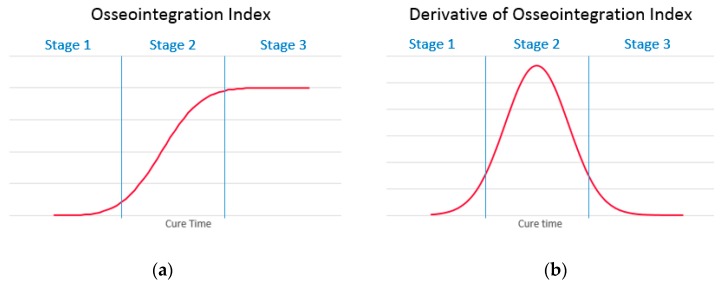
(**a**) Osseointegration Index and (**b**) its time derivative of an ideal model.

Previous studies have discussed the healing characteristics of a fractured bone, which is associated with the increase of whole-bone stiffness [33,77,78]. It is anticipated that the osseointegration process trend is similar to fractured bone healing. The O-Index is categorised into three stages (refer to Figure 3): First stage (early osseointegration stage) where the gradient increases; Second stage (where the implant is osseointegrating with the parent bone) highest gradient hence point of inflection; and last stage (where the implant/bone is fully osseointegrated) gradient decrease and signal plateaus. Additionally, the time derivative of O-Index is an essential supplementary metric that helps identify the point of inflection and the stages.

This study presents acousto-ultrasonic stress wave interrogation technique to measure osseointegration levels of a bone and implant for health monitoring and assessment. The frequency analysis is conducted to investigate and discuss the wave propagation in the construct as the bone-implant osseointegrates. Furthermore, O-Index is calculated for each specimen to assess with the definition of a numerical quantity that can be used to describe the state of osseointegration.

## 3. Methods

### 3.1. Specimens

**Figure 4:**
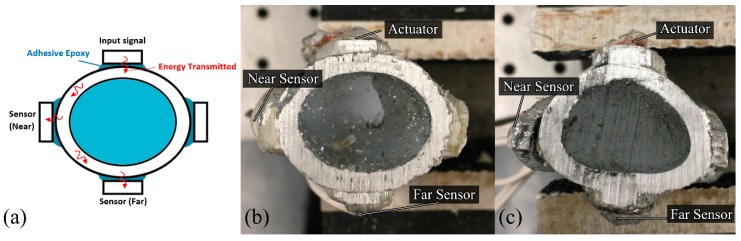
(**a**) Schematic of sensors location and specimen bonded with adhesive epoxy; Oval (**b**) and triangular (**c**) cross-section aluminium specimen with 4-extramedullary struts design, including near and far sensor placements on the struts.

Two 3 mm thick aluminium 6060 T5 mill-finished cylinders with different diameters are used for this investigation. A small cylinder of outer diameter 25 mm approximately 130 mm in length is used as the shape-like bone structure. Since the cross-sectional bone structure is different for each amputated patient, refer to Figure 1, oval and triangular-like aluminium surrogates are produced. A large cylinder of outer diameter 32 mm is used to make two 4-extramedullary strut implant based on the novel osseointegration implant design [2], refer to Figure 2. Both cylinders are pressed to shape to produce the aluminium surrogate bone and snug-fitted surrogate implant with the oval and triangular cross-sections (refer to Figure 4). In this study, the aluminium surrogate implant has four extramedullary struts, which extend approximately 50 mm in length and the case depth of approximately 15 mm. The distance between the extramedullary struts is approximately 22 mm (at the tip). A rubber back cover is adhesively bonded to constraint translational movement along the cylinder.

The curing of the adhesive epoxy, which has been used to model the healing of a fractured bone, is used to simulate the osseointegration process in this study [23,76,79,80,81]. In a physiological perspective, the osseointegration process begins from a blood clot to callus mineralisation and ossification which is primarily similar to the curing process of viscous elastic epoxy from liquid to solid state [82,83]. The use of epoxy for curing is not an exact representation for osseointegration. Furthermore, the solidified epoxy properties (Young’s Modulus and Poisson’s ratio) are not equivalent to the parent material properties. However, the curing process is similar to an extent to fundamentally demonstrate the concept of changes in material properties.

The experimental work involves three piezoelectric elements (i.e., an actuator and two receiver sensors) bonded onto the different struts of the implant to measure the frequency responses as the specimen cures (refer to Figure 4). The actuator excites; transmit energy, in the form of stress waves, and as the specimen unifies, the energy transmission changes due to the change in stiffness of the overall construct. Our future investigation on continuous osseointegration monitoring will consider the intramedullary stem and other possible locations for sensors placement.

The effect of soft tissues on the dynamic response of a human tibia has been reported that the mass-loading, predominantly due to the muscle, decreases the resonant frequency and increase damping [84,85,86,87,88]. In order to simulate the soft tissues effects, plasticine; modelling clay, is used to surround the whole specimen to simulate the soft tissue mass-loading and damping. Plasticine is chosen because of its high damping quality, and it can be moulded to ensure maximal contact, easily removed and re-used. The inside of surrogate bone sections are filled with silicone and cured for 48 h to simulate the soft cancellous bone in the medullary cavity.

### 3.2. Experimental Setup

PZT Pz26 (Meggitt PLC, Bournemouth Airpot, Dorset, UK) of 5 mm diameter and 2 mm thickness is used as the actuator and Pz27 of 5 mm diameter and 1 mm thickness is used as the receiver. The electronic transducers are bonded onto the tip of the extramedullary struts as a shown in Figure 4. The two receivers are bonded on the strut: one closest to the actuator approximately ¼ circumference away (~25 mm); as the near sensor, and one furthest to the actuator approximately ½ circumference away (~50 mm); as the far sensor, for each specimen, refer to Figure 4.

The actuator excites a 50 V 1 MHz triangular pulse signal which is generated by the NI PCI5412 Function Generator (National Instruments, Austin, TX, USA). The input signal is filtered in a Model 3944 multichannel filter (Krohn-Hite, Brockton, MA, USA) and amplified by a Krohn-Hite Model 7602 wideband amplifier. The raw output signals are recorded using PicoScope 6402D and its software (Pico Technology, Cambridgeshire, UK) with a sample interval setting of 2 ns for a total of 50,000 samples with 128 averages.

**Figure 5:**
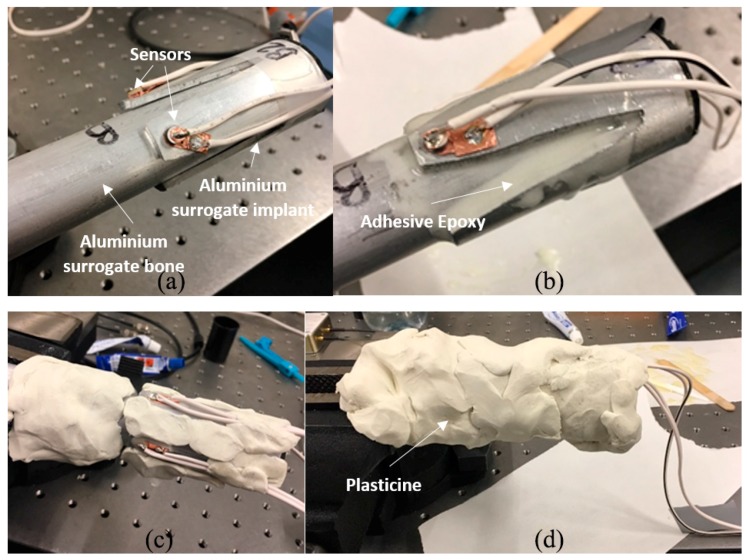
Images of the specimen with adhesive epoxy and plasticine: (**a**) Aluminium surrogate implant snug-fit with aluminium surrogate bone cylinder; (**b**) Specimen with adhesive applied between the aluminium surrogate implant and bone; (**c**) Specimen partially covered with plasticine prior to the experiment and (**d**) Specimen fully covered with plasticine.

Prior to the experiment, the specimen is first partially surrounded by plasticine. Two-hour epoxy (Bostik, Milwaukee, WI, USA) (work time of 2 h and maximum full cure after 16 h) is applied between the aluminium surrogate implant and bone, then immediately the whole specimen is fully covered by plasticine and the time output signal starts recording, refer to Figure 5. Cure time, T, is the time measurement of the epoxy curing. The total duration of 1000 min cure time is recorded: the first 30 min at 1-min increment and afterwards at 5-min increments. Each specimen mass is measured and tabulated in Table 1. It is evident that the test specimens are severely mass-loaded.

**Figure 6:**
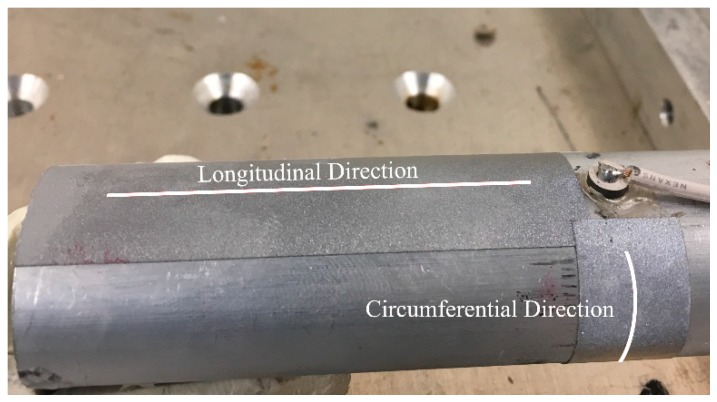
Image of oval surrogate bone with showing the longitudinal and circumferential scan line.

**Figure 7:**
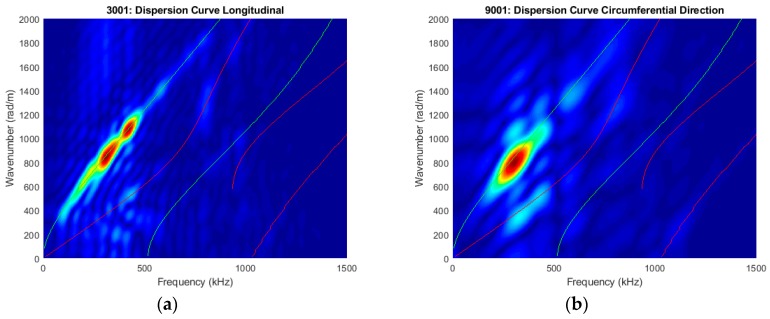
Experimental dispersion curves of a 1 MHz triangular pulse in the (**a**) longitudinal and (**b**) circumferential directions.

It should be noted that the circumferential guided wave modes for a traction-free cylinder are dispersive and its dispersion curve is very similar to the Lamb wave mode (guided waves in plates) when the radius to thickness ratio is large and cylindrical thickness is small [40,89,90,91,92]. The excited signal mainly consists of fundamental wave modes at this frequency-thickness excitation. In the preliminary study, the Pz26 actuator was bonded on the oval surrogate bone and in order to verify the excitation modes, out-of-plane laser vibrometry (POLYTEC Inc., Irvine, CA, USA) was used to perform longitudinal and circumferential line scans at time sampling of 0.1µs and scan length of 35 mm, starting 10 mm away from the actuator, refer to Figure 6. Retroreflective films were attached to the line scans to enhance the signal to noise ratio. For the longitudinal scan, 128 spatial points were recorded and displaced automatically using the XY positioning system. For the circumferential scan, 35 points were recorded and displaced manually by rotating the surrogate bone. However, the curvature of the oval surrogate was not entirely perpendicular to the laser head. As a result, the recorded signals are not entirely in the out-of-plane direction (normal to the curvature), and consequentially, the fundamental symmetric mode is more apparent in the circumferential direction refer to Figure 7. Nevertheless, this technique serves to identify the propagating guided wave modes.

In our previous FE study on stress wave-based monitoring on novel endoprosthesis design [72], a 130 mm long hollow aluminium cylinder of 25 mm outer diameter and 3 mm thickness was modelled as the surrogate bone. The FE dispersion curve of the cylinder circumference indicated a dominant fundamental circumferential antisymmetric (flexural) guided-wave for a 1 MHz triangular pulse excitation. The dispersion curves measured (as shown in Figure 7) substantiated the FE study [54]. Two-dimensional fast Fourier transformation is performed on the points along the longitudinal and circumferential lines to create the dispersion curves in order to identify the dominant guided wave modes from DISPERSE [90]. The experimental results show that the circumferential wave propagating on the triangular and oval cross-section would be similar to those travelling around the cylinder circumference as shown in the dispersion curves in Figure 7. The dominant wave mode is the flexural circumferential wave modes in both directions, and a weak symmetrical wave mode is also present [90].

Since the core of the specimen is filled with silicone rubber, the wave propagation characteristics across this material were also determined. Blocks of silicone rubber with different dimensions were constructed, and the average longitudinal bulk wave velocity of 1098.9 m/s was determined using pulse-echo method and V539 transducers (Panametrics, Waltham, MA, USA).

To assist with the wave mode identification in a transient time-frequency domain, MATLAB R2017b (MathWorks, Natick, MA, USA) is used to develop a spectrogram to visualise the transient power spectral density (PSD) of the acousto-ultrasonic signal received by the sensing element. The dominant wave modes in the spectrograms presented in the following sections are identified based on the experimental group velocities, arrival time and the possible wave paths, which include the shortest and longest circumferential path from the actuator to the near and far sensors, the longitudinal path from the ends of the specimens, and the through-silicone-core path.

## 4. Experimental Results

### 4.1. Specimens with Oval Cross-section

**Figure 8:**
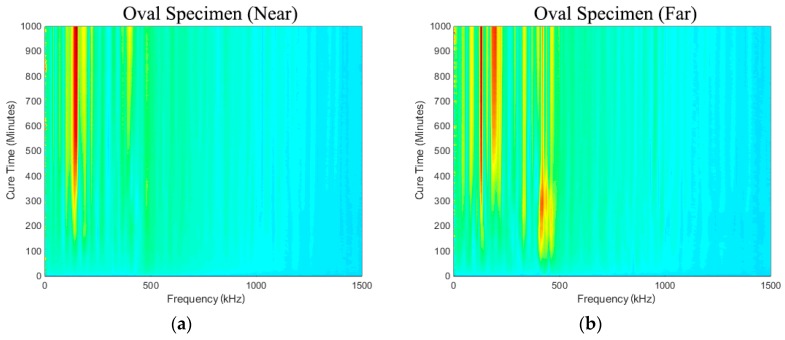
Oval specimens (**a**) Near and (**b**) Far: Change in frequency spectrum for each cure time relative to the baseline.

Figure 8a,b show the spectra development as a function of cure time (i.e., simulated osseointegration). It is evident that response measured in the frequency range from 50 kHz to 500 kHz increases throughout cure time. The sensitivity of the early curing is noticeable after approximately 300 min, where the response in the frequency band approximately 130~250 kHz increased significantly. This is consistent with the 2-h work time of the adhesive used.

**Figure 9:**
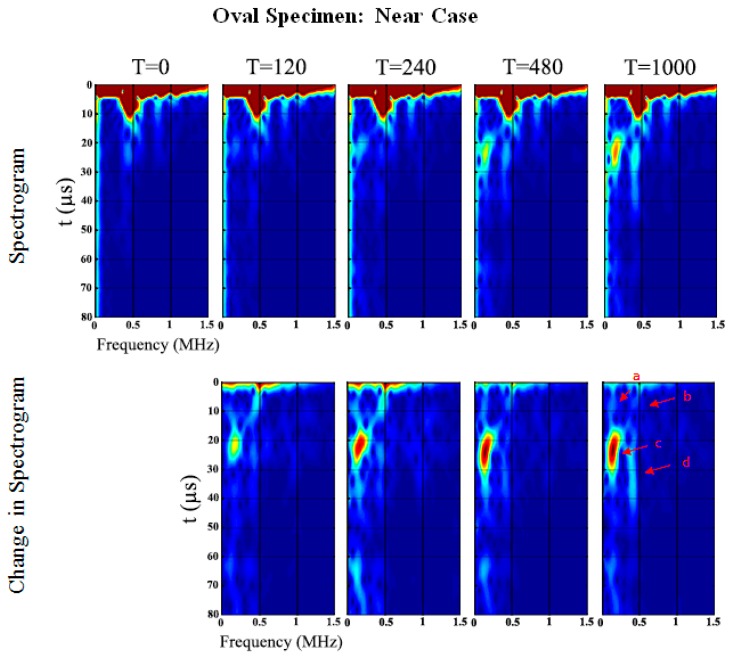
Oval specimen (Near): Spectrogram and changes in spectrogram for each cure time relative to the baseline.

**Figure 10:**
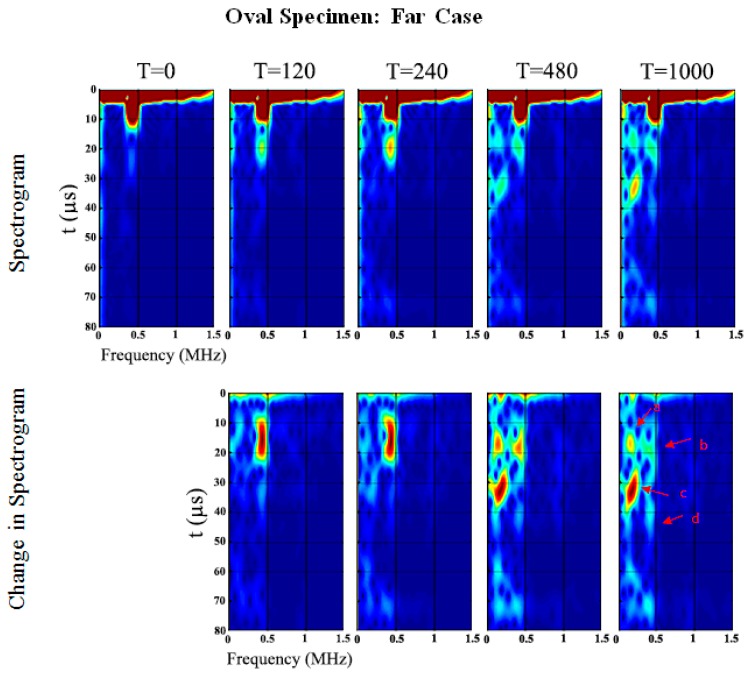
Oval specimen (Far): Spectrogram and changes in spectrogram for each cure time relative to the baseline.

The spectrograms of the time-series at five different stages (0, 120, 240, 480 and 1000 min) of cure time are shown in Figure 9 and Figure 10 for the ‘near’ and ‘far’ sensors, respectively (see also Figure 4). In the oval specimen near-case, two waves at low and high frequencies propagate around the specimen circumference from both directions. The circumferential flexural waves propagating from the shortest distance arrives at 9 µs for 99.2 kHz (Wave *a*) and 6.4 µs for 465.4 kHz (Wave *b*), refer to Figure 9 and Table 2. Waves *c* and *d* arrive at 21.1 µs for 167.8 kHz and 22.1 µs for 434.9 kHz, respectively. It should be noted that the longitudinal flexural waves reflected at the ends of the specimens arrive slightly after the circumferential flexural wave propagating from the longest distance, which serves the explanation of the larger spread of Waves *c* and *d* in time domain (refer to Figure 9). The frequency of the ultrasonic guided wave Wave *a* increases, however, the others decrease as cure time increases. Furthermore, Wave *c* significantly increases in PSD magnitude of 27.2 dB/Hz from cure time 0 min. Wave *b* slightly increases in PSD magnitude of 10.5 dB/Hz at cure time 240 min however it slowly decreases afterwards to a change in PSD magnitude of 0.1 dB/Hz at cure time 1000 min.

In the oval specimen far-case (refer to Figure 10 and Table 3), the flexural waves propagating in both directions arrive at the same time since the sensor is placed an almost equal distance from the actuator. The first arriving circumferential flexural waves, Waves *a* and *b*, are observed to be developing from 0 to 240 min cure time. Afterwards, Waves *c* and *d*, identified as the returning longitudinal flexural waves reflected at the ends of the specimens, are more apparent.

Similar to the near-case, the frequency of the ultrasonic guided wave Wave *a* increases while the others decrease in frequency as cure time increases, see Figure 10 and Table 3. Wave *c* in the far-case also significantly increases in PSD magnitude of 27.6 dB/Hz as cure time increases. Wave *b* increases in PSD magnitude of 11.5 dB/Hz at cure time 240 min then decreases to 9.8 dB/Hz at cure time 1000 min.

### 4.2. Triangular Specimen

**Figure 11:**
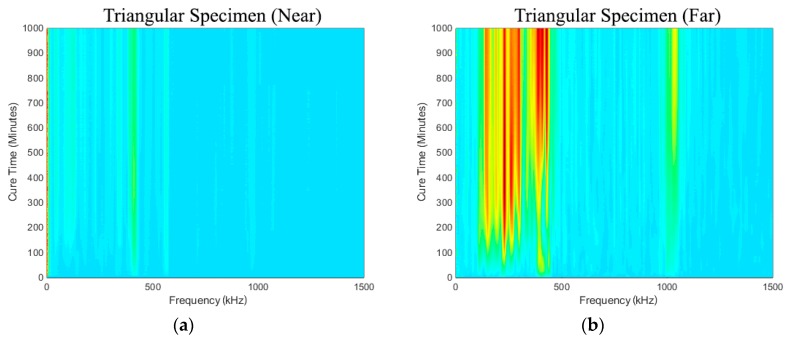
Triangular specimen (**a**) Near and (**b**) Far: Change in frequency spectrum for each cure time relative to the baseline.

Figure 11a,b show the spectra development as a function of cure time. As in the results presented in the previous section, it is evident that the frequency range from 50 kHz to 500 kHz increases throughout cure time. The sensitivity of the early curing is noticeable after 300 min, where the frequency of approximately 130~250 kHz increased significantly. However, the triangular specimen near-case response, shown in Figure 11a, is noticeably different from the others, and there is no evidence of the appearance of any significant frequency peak or prominent change that is similar to the others.

**Figure 12:**
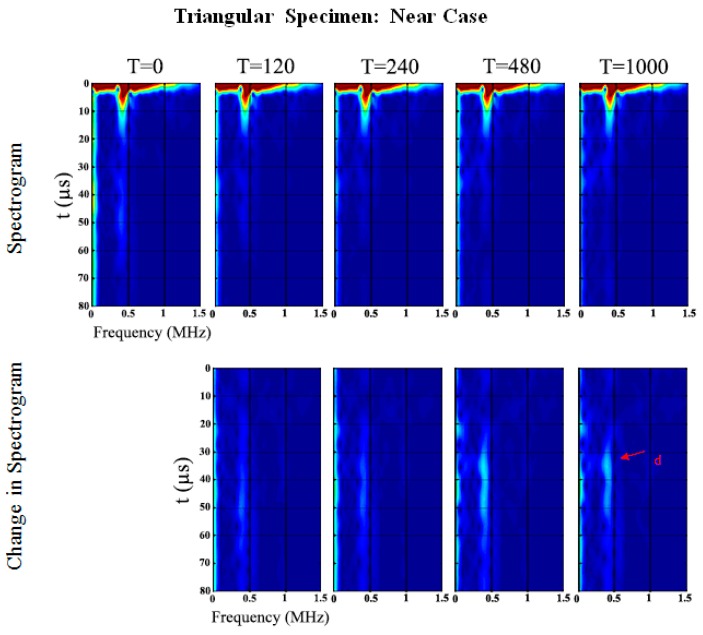
Triangular specimen (Near): Spectrogram and changes in spectrogram for each cure time relative to the baseline.

**Figure 13:**
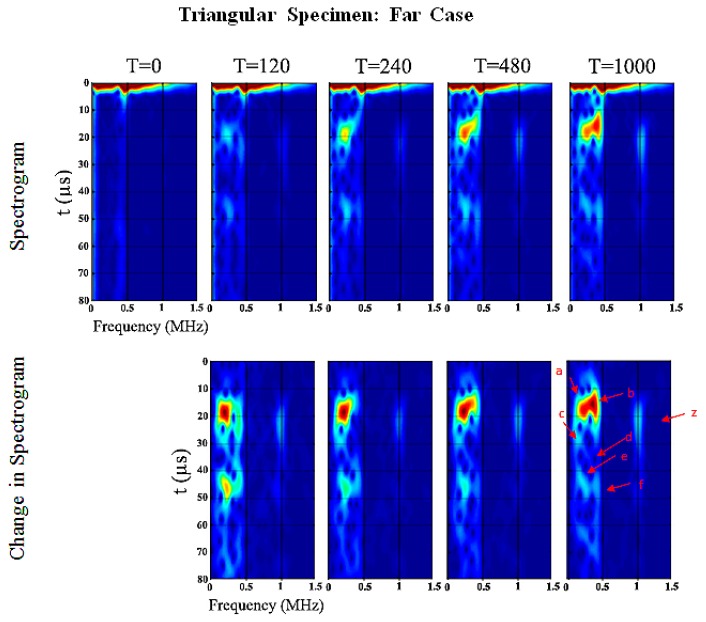
Triangular specimen (Far): Spectrogram and changes in spectrogram for each cure time relative to the baseline.

Similar to the oval specimen, Figure 12 and Figure 13 show the spectrogram at the five different stages of cure time. In the triangular specimen near-case, only one change in frequency of 389.1 kHz at 36 µs which shifts to 381.5 kHz at 38.1 µs and decreases in PSD magnitude of 6.1 dB/Hz as cure time increases, refer to Table 4 and Figure 12. Wave *d* is identified as the returning longitudinal flexural wave mode reflected at the end of the specimen. The results are distinctly different from the other cases presented in this paper. The significance of this set of results will be discussed later.

Figure 13 shows the spectrogram of the triangular specimen far-case. As in previous oval specimen results, the development of the spectral response as a function of cure time is pronounced. Wave *e* and *f* are the returning circumferential flexural waves of Wave *a* and *b*, respectively. Waves *c* and *d* are the returning longitudinal flexural waves reflected at the ends of the specimens. Wave *a* increases in frequency whereas Wave *b*–*f* decrease and Wave *z* remains the same, refer to Table 5 and Figure 13. Wave *a* and *b* change in PSD magnitude increase significantly of 29.5 dB/Hz and 26.9 dB/Hz as cure time increases, respectively. Furthermore, Wave *z* is one of the higher-order modes and unfortunately it is difficult to identify in this frequency range. This result will be discussed in the following section which shows that the changes in the energy of the signal are a good indication of the degree of osseointegration.

## 5. Osseointegration Index

**Figure 14:**
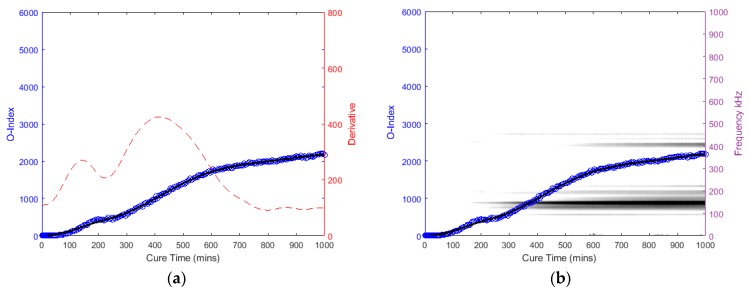
Oval specimen (Near): O-Index with (**a**) its time derivative and (**b**) its change in frequency.

**Figure 15:**
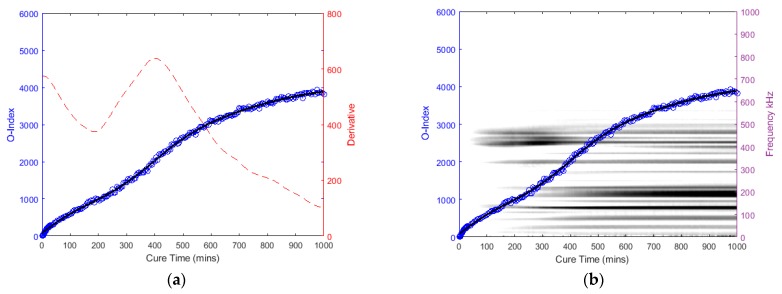
Oval specimen (Far): O-Index with (**a**) its time derivative and (**b**) its change in frequency.

**Figure 16:**
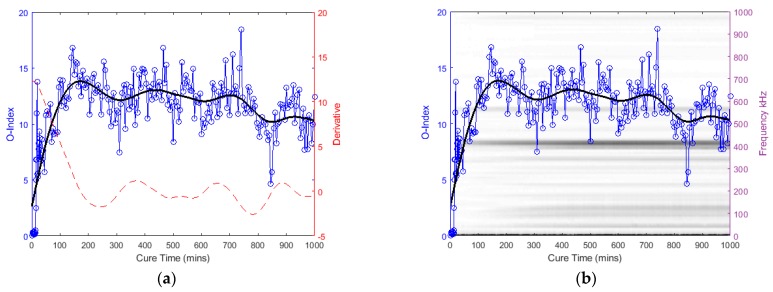
Triangular specimen (Near): O-Index with (**a**) its time derivative and (**b**) its change in frequency.

**Figure 17:**
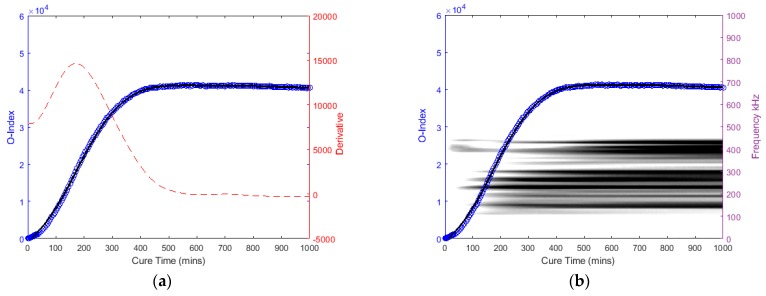
Triangular specimen (Far): O-Index with (**a**) its time derivative and (**b**) its change in frequency.

The following graphs in Figure 14, Figure 15, Figure 16 and Figure 17 for the specimens with oval and triangular cross-sections, respectively, were produced by the O-Index formula. It is shown that the O-Index steadily increases over cure time and later plateaus; gradient approaching zero, as it fully bonds with the surrogate implant. The curing of the adhesive resulted in an initial high gradient as seen in Figure 14, Figure 15 and Figure 17. In Figure 14 and Figure 15, the maxima gradients are located approximately at 400 min, whereas, in Figure 17, the triangular specimen far-case the maxima gradient is located approximately 187 min.

The O-Index magnitude of the oval specimen cases is relatively similar however the triangular specimen far-case is an order of magnitude greater than the oval specimen. The increase and decrease in energy for the oval specimen at approximately 400~450 kHz; Label *b* (refer to Figure 9)*,* has influenced the O-Index in the early stages as seen from the time derivative. Overall, the O-Indices are primarily driven by the change in frequency between 100 kHz to 300 kHz, refer to Figure 14b, Figure 15b and Figure 17b. The results for both cases for the oval specimen and far-case for the triangular specimen have shown a clear increasing asymptotic trend in the later stages. However, triangular specimen near-case did not show similar O-Index and has different magnitude, trend, and oscillating gradient (refer to Figure 16).

## 6. Discussion

**Figure 18:**
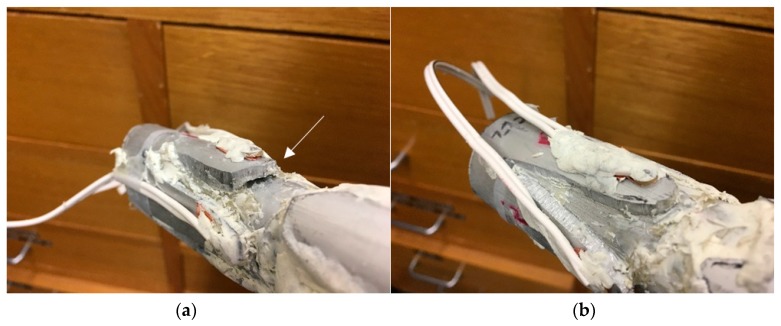
Triangular cross section of (**a**) Near: inadequate bonding and (**b**) Far: successfully bonding.

After the experimental investigation, the plasticine was removed to allow visual inspection of the adhesive layer between the implant and bone cylinder. Whilst the extramedullary struts of the oval section specimen was fully and successfully bonded to the aluminium surrogate bone (i.e., fully osseointegrated), the near strut of the triangular specimen did not integrate adequately (i.e., absence of osseointegration), leaving an approximately 10 mm depth gap, due to lack of epoxy adhesive between the specimen interfaces, refer to Figure 18. This inadequate bonding can be interpreted as a lack of or absence of osseointegration. Furthermore, this serves as an explanation to the triangular specimen near-case result: absence of inflection point and noisier compared to the unified cases. In comparison, it is worth noting that successfully bonded cases showed significant changes in the low frequencies between 100~300 kHz. In this regard, it is evident that the proposed assessment methodology described above can be used to assess the degree and the lack of osseointegration.

One would expect the first arriving wave mode is the most predominant feature as shown in the triangular specimen far-case, however, the oval specimens have shown a significant increase of a later arriving wave and an earlier increase in unification prior to the inflection. This can be attributed to the fact that the reaction of adhesive epoxy begins immediately upon mixing the two components and the portions are not controlled. This gives rise to variation in fully cure duration and hardening. The adhesive layer may not have been uniform and consequentially, uneven curing at different areas of the specimen. The preparation time taken to assemble the test specimen upon the application of the adhesive and the installation of the plasticine to simulate soft tissue damping is also different in each specimen. Nevertheless, these features indicated that the O-Index is sensitivity to the bonding quality between the implant and bone structure. It is also evident that the inclusion of the damping effects by silicone and the highly damped plasticine did not affect the ability of the proposed osseointegration assessment methodology.

The osseointegration phenomena is a complex process with viscoelastic, anisotropic, and heterogeneous properties, and additionally, bone remodelling phenomena and other biological factors will influence the O-Index. The complex geometry of bone and soft tissue damping are known common problems which hinder the applicability of assessment technique adoption for clinical use. The effect of changing environmental and operational constraints can be a significant influence as it may decrease sensitivity and lead to false-positive indication [93,94]. It is known that the variation of sensor and bonding properties becomes significant due to, primarily, temperature effect, influencing the measurements of guided waves [55,56,94,95]. Recent works have shown that these effects can be compensated for baseline methods in the time domain [55,56,96,97]. It is difficult to distinguish other secondary influences and its effect on the ultrasonic wave reading in the human body until appropriate trials are conducted. In addition, the sensors material biocompatibility, its ability to conform to bone-implant geometry, and sensor placements are also the additional challenges when incorporating sensing systems to implant design for continuous assessment. Nevertheless, further advancement of clinical studies is required to sequentially scrutinise the concerns and factors in order to develop a robust bone-implant osseointegration monitoring system.

Our future work will include detailed FE investigation including modelling and representative substitution for soft tissue, composite bone model and implant material to further validate this acousto-ultrasonic method for potential continuous monitoring and assessment of osseointegration. A quantitative measurand will potentially assist in identifying and predicting common implant failures and complications, such as construct failure, aseptic loosening, and skin-implant infection, consequently prompting early rehabilitation and body functionality.

## 7. Conclusions

An acousto-ultrasonic stress wave technique to analyse the frequency response over cure time has been demonstrated to assess and monitor integrating of a bone-like and implant surrogate. The findings indicated that O-Index provides a plausible approach for continuous monitoring of the degree of osseointegration. The spectrogram indicated changes in the low-frequency response at different arrival times as the specimen cures. It is shown that the development of low-frequency throughout cure time indicates successfully bonding between the aluminium surrogate implant and bone. The O-Index trend and its derivative can be used to identify the different stages and absence of osseointegration. Future work is currently underway to investigate this acousto-ultrasonic method in clinical/animal trials and to optimise on novel implant design.

## Figures and Tables

**Table 1:** Oval and triangular cross-section specimens’ masses.

Cross-section	Aluminium Surrogates (Implant and Bone) Mass (grams)	Plasticine Mass (Grams)
Oval	523.5	317.9
Triangular	504.1	299.0

**Table 2:** Oval Specimen (Near): Wave information.

Wave	Frequenc (0 min)	Time (0 min)	Frequency (1000 min)	Time (1000 min)	Change in Power/Frequency (dB/Hz) (relative to 0 min)
*A*	99.2 kHz	9 µs	137.3 kHz	7.6 µs	7.8 dB/Hz
*B*	465.4 kHz	6.4 µs	465.4 kHz	6.5 µs	0.1 dB/Hz
*C*	167.8 kHz	21.1 µs	145.0 kHz	23.7 µs	27.2 dB/Hz
*D*	434.9 kHz	22.1 µs	404.4 kHz	25.7 µs	6.7 dB/Hz

**Table 3:** Oval specimen (Far): Wave information.

Wave	Frequency (0 min)	Time (0 min)	Frequency (1000 min)	Time (1000 min)	Change in Power/Frequency (dB/Hz) (relative to 0 min)
*a*	76.3 kHz	18.6 µs	152.6 kHz	16.6 µs	18.6 dB/Hz
*b*	419.6 kHz	19.3 µs	381.5 kHz	19.7 µs	9.8 dB/Hz
*c*	198.4 kHz	32.3 µs	183.1 kHz	33.2 µs	27.6 dB/Hz
*d*	434.9 kHz	36.4 µs	412.0 kHz	40.2 µs	22.5 dB/Hz

**Table 4:** Triangular specimen (Near): Wave information.

Label	Frequency (0 min)	Time (0 min)	Frequency (1000 min)	Time (1000 min)	Change in Power/Frequency (dB/Hz) (relative to 0 min)
*d*	389.1 kHz	36 µs	381.5 kHz	38.1 µs	−6.1 dB/Hz

**Table 5:** Triangular specimen (Far): Wave information.

Label	Frequency (0 min)	Time (0 min)	Frequency (1000 min)	Time (1000 min)	Change in Power/Frequency (dB/Hz) (relative to 0 min)
*a*	244.1 kHz	21.2 µs	251.8 kHz	18.0 µs	29.5 dB/Hz
*b*	419.6 kHz	23.4 µs	381.5 kHz	15.7 µs	26.9 dB/Hz
*c*	198.4 kHz	28.8 µs	160.20 kHz	28.7 µs	22.2 dB/Hz
*d*	373.8 kHz	36.3 µs	343.3 kHz	37.1 µs	18.0 dB/Hz
*e*	244.1 kHz	47.2 µs	228.9 kHz	44.5 µs	24.0 dB/Hz
*f*	427.2 kHz	47.8 µs	419.6 kHz	47.6 µs	11.1 dB/Hz
*z*	1030.0 kHz	21.5 µs	1030.0 MHz	21.6 µs	22.4 dB/Hz
